# High field MR carotid vessel wall imaging: reproducibility of five different MR-weightings

**DOI:** 10.1186/1532-429X-13-S1-O13

**Published:** 2011-02-02

**Authors:** Eleanore S Kroner, Jos J Westenberg, Rob J van der Geest, Joost Doornbos, Joanne D Schuijf, Eline Kooi, Albert de Roos, Jeroen J Bax, Hildo J Lamb, Hans-Marc Siebelink

**Affiliations:** 1 LUMC, Leiden, Netherlands; 2 AZM, Maastricht, Netherlands

## Introduction

Magnetic Resonance Imaging (MRI) has emerged as a promising noninvasive imaging modality for the serial assessment of vessel wall thickness in the carotid artery as an early marker of atherosclerosis. For clinical application of this technique, Scan-Rescan reproducibility is paramount. Currently, a multicontrast protocol, including a combination of MR-weightings is used as reference standard for quantitative and morphologic measurements.

## Purpose

To investigate Scan-Rescan reproducibility for each of the commonly used weightings analyzed separately. To investigate which of the MR-weightings approximates best the combined multicontrast protocol (reference standard).

## Methods

**Figure 1:**
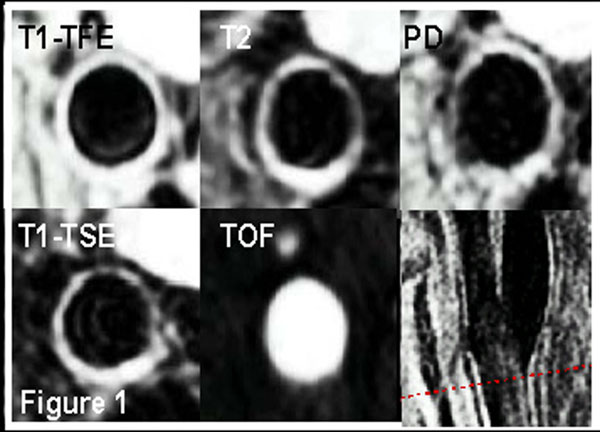
An example of the co-registered contrast-weightings and 3D time-of-flight. The red line on the sagittal view of the carotid bifurcation indicates position of the analyzed slices.

5 healthy volunteers (60% male, mean age=28years) underwent repeated MRI examinations of the left carotid artery with five contrast-weighted scans to image lumen and vessel wall (Table 1). The scan and a rescan were acquired using a 3T (Philips) MRI scanner. A standard phased-array coil with two flexible elements of 14×17cm was used to obtain nine transverse imaging sections of the left carotid artery with identical in-plane resolution (0.46×0.46mm^2^). Scan-Rescan analysis was performed in the third slice of the imaging stack, representing a slice in the common carotid artery. An example is provided in Figure 1. Manual contour segmentation of the lumen and vessel wall was performed using in-house developed software (VesselMASS). Vessel wall area (mm^2^) and lumen area (mm^2^) were assessed by one blinded observer for the different contrast weightings and compared with the rescan acquisition. Furthermore, vessel wall- and lumen areas from the different contrast weightings were compared with the reference standard.

**Table 1:** Carotid Imaging Protocol at 3T: Scan Parameters

Parameters	Black-blood T1-weighted	Black-blood T2-weighted	Black-blood Proton-Density-weighted	T1-weighted	TOF
Acquisition sequence	TFE	TSE	TSE	TSE	FFE
Acquisition Mode	2D	2D	2D	2D	3D
Echo Time (msec)	3.54	50	20	10	3.30
Repetition Time (msec)	12.41	2 heartbeats	2 heartbeats	1 heartbeat	26.20
Excitation flip angle (degrees)	45	90	90	90	20
FOV (cm)	14 x 14	14 x 14	14 x 14	14 x 14	14 x 14
Resolution (mm2)	0.461 x 0.461	0.461 x 0.461	0.461 x 0.461	0.461 x 0.461	0.461 x 0.461
Slice thickness/gap (mm)	2; 0.71	2; 0.71	2; 0.71	2; 0.71	2; 0.71
Slices	9	9	9	9	9

TSE, turbo (segmented) spin-echo; FFE, fast field echo (gradient echo); TFE, turbo field echo; FOV, Field of view; TOF, time of flight

## Results

**Figure 2:**
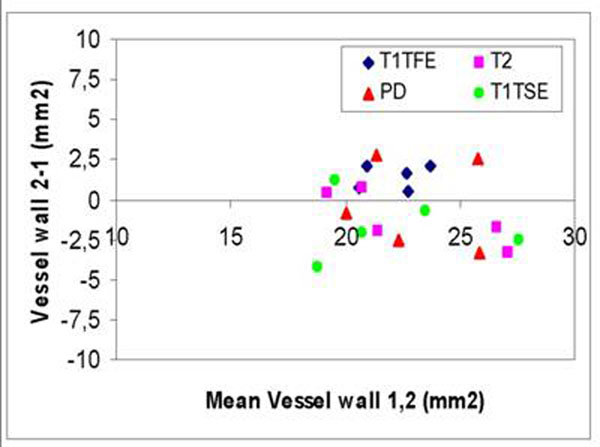
Bland-Altman Plot: Scan-Rescan Reproducibility (Vessel Wall Area)

Reproducibility of the repeated assessment was high for all MR-weightings, for both lumen area and vessel wall areas. (Table 2). The Bland-Altman plot for vessel wall area, is shown in Figure 2 for Scan-Rescan reproducibility. Highest reproducibility was found for the T1-TFE and T2-TSE sequences.

**Table 2:** 

a- Scan-Rescan Reproducibility for the Common Carotid Artery											
	Lumen Area (mm2)						Vessel Wall area (mm2)				

	R	mean	p	SD	COV (%)		R	mean	p	SD	COV (%)

T1-TFE	0.97	-1.15	0.42	2.91	8%		0.86	1.41	0.01	0.74	3%
T2	0.95	2.03	0.18	2.82	8%		0.96	-1.19	0.20	1.72	7%
PD	0.89	2.75	0.06	2.28	7.5%		0.55	-0.25	0.85	2.82	12%
T1-TSE	0.90	-0.12	0.94	3.45	10%		0.85	-1.70	0.13	2.03	9%
TOF	0.97	0.74	0.43	1.89	5%						

b- Separate analysis of the MR-weightings compared to combined contrast weighted protocal											

	Lumen Area (mm2)						Vessel Wall area (mm2)				

	R	mean	p	SD	COV (%)		R	mean	p	SD	COV (%)

T1-TFE	0.97	0.57	0.52	1.80	5%		0.90	-0.60	0.18	0.83	3.5%
T2	0.38	-0.76	0.85	8.32	24%		0.52	-1.02	0.42	2.54	11%
PD	0.81	-2.71	0.24	4.39	13%		0.07	-0.50	0.75	3.31	14%
T1-TSE	0.78	-0.69	0.75	4.63	13%		0.64	-2.25	0.15	2.87	13%
TOF	0.84	3.08	0.15	3.88	10%						

R. Pearson’s correlation coefficient; P p-value of T-test; SD. Standard deviation; COV. Coefficient of variation

T1-TFE showed highest correlation for lumen (r=0.97) and vessel wall area (r=0.90) assessment when compared with the reference standard.

## Conclusion

This pilot Scan-Rescan study showed best reproducibility of lumen and vessel wall area assessment for the T1-TFE and T2-TSE weightings. T1-TFE showed highest correlation to the reference standard.

